# Association between genetically plasma proteins and osteonecrosis: a proteome-wide Mendelian randomization analysis

**DOI:** 10.3389/fgene.2024.1440062

**Published:** 2024-07-25

**Authors:** Chen Meng, Junxiao Ren, Honglin Gu, Hongxin Shi, Huan Luo, Zhihao Wang, Chuan Li, Yongqing Xu

**Affiliations:** ^1^ School of Graduate, Kunming Medical University, Kunming, Yunnan, China; ^2^ Department of Orthopaedic, 920th Hospital of Joint Logistics Support Force of Chinese People’s Liberation Army, Kunming, Yunnan, China; ^3^ The First School of Clinical Medical, Yunnan University of Chinese Medicine, Kunming, Yunnan, China; ^4^ Department of Spine Surgery, Guangdong Provincial People’s Hospital (Guangdong Academy of Medical Sciences), Southern Medical University, Guangzhou, China; ^5^ Kunming Institute of Zoology, Chinese Academy of Sciences, Kunming, Yunnan, China

**Keywords:** Mendelian randomization, plasma proteins, osteonecrosis, genome-wide association studies, HEBP1

## Abstract

**Background:**

Previous studies have explored the role of plasma proteins on osteonecrosis. This Mendelian randomization (MR) study further assessed plasma proteins on osteonecrosis whether a causal relationship exists and provides some evidence of causality.

**Methods:**

Summary-level data of 4,907 circulating protein levels were extracted from a large-scale protein quantitative trait loci study including 35,559 individuals by the deCODE Genetics Consortium. The outcome data for osteonecrosis were sourced from the FinnGen study, comprising 1,543 cases and 391,037 controls. MR analysis was conducted to estimate the associations between protein and osteonecrosis risk. Additionally, Phenome-wide MR analysis, and candidate drug prediction were employed to identify potential causal circulating proteins and novel drug targets.

**Results:**

We totally assessed the effect of 1,676 plasma proteins on osteonecrosis risk, of which 71 plasma proteins had a suggestive association with outcome risk (*P* < 0.05). Notably, Heme-binding protein 1 (HEBP1) was significant positively associated with osteonecrosis risk with convening evidence (OR, 1.40, 95% CI, 1.19 to 1.65, *P* = 3.96 × 10^−5^, *P*_FDR_ = 0.044). This association was further confirmed in other MR analysis methods and did not detect heterogeneity and pleiotropy (all *P* > 0.05). To comprehensively explore the health effect of HEBP1, the phenome-wide MR analysis found it was associated with 136 phenotypes excluding osteonecrosis (*P* < 0.05). However, no significant association was observed after the false discovery rate adjustment.

**Conclusion:**

This comprehensive MR study identifies 71 plasma proteins associated with osteonecrosis, with HEBP1, ITIH1, SMOC1, and CREG1 showing potential as biomarkers of osteonecrosis. Nonetheless, further studies are needed to validate this candidate plasma protein.

## Introduction

Osteonecrosis, often referred to as ischemic necrosis, aseptic necrosis, or avascular necrosis, typically manifests as osteonecrosis of the femoral head (ONFH) (Chang et al., 2020). Approximately 20,000 new cases of osteonecrosis are diagnosed in the United States (Hungerford, 2002), with a cumulative patient count of ONFH ranging from 300,000 to 600,000 (Petrigliano and Lieberman, 2007). In advanced stages of ONFH, subchondral bone collapse and progressive hip joint deterioration can occur, leading to a loss of work capacity for the patient and substantial financial strain on the families. If left untreated, osteonecrosis can lead to future disability (Malizos et al., 2007) Current diagnosis primarily relies on X-ray and MRI, yet there are no validated clinical biomarkers to identify osteonecrosis of activity, turnover, and prognosis. Given the significant socioeconomic burden and the scarcity of effective treatments, there is a pressing need to further identify biomarkers with potentially possible diagnostic or prognostic value for osteonecrosis.

Proteomics is crucial in clinical diagnostics and monitoring, showing the ability to discover novel proteins in diseased tissues, biological fluids, and serum. Previous studies indicate that certain proteins may contribute to osteonecrosis development and offer therapeutic benefits (Tan et al., 2006; Chen et al., 2015; An et al., 2021). For instance, serum levels of tissue plasminogen activator (t-PA), plasminogen activator inhibitor-1 (PAI-1), CrossLaps, and anti-p53 antibody have been identified as potential noninvasive diagnostic biomarkers for ischemic osteonecrosis of the femoral head (IONFH) (Tan et al., 2006). Another study observed significantly reduced serum levels of complement component 3 (C3), C4, inter-α-trypsin inhibitor heavy chain H4, and α-2-macroglobulin in steroid-induced ONFH patients (Chen et al., 2015). In addition, animal studies have shown that Nel-like protein-1 has pro-angiogenic and osteogenic effects (An et al., 2021). However, these studies are primarily observational and susceptible to confounding factors and reverse causation. Additionally, with the development of genome-wide association studies (GWAS) at the levels of circulating protein, sequence determinants of protein levels (pQTLs), helped to identify causative genes and elucidate disease pathways.

Mendelian randomization (MR) utilizes genetic variants as instrumental variables, reducing susceptibility to confounders since these variants are randomly assigned at conception, independent of environmental and individual characterisrics. A complete investigation into the causal effect of plasma proteins on disease has been made possible by the large-scale integration of the plasma proteome with genetics and disease in large samples (Ferkingstad et al., 2021; Geyer et al., 2021). In this study, we utilized MR analysis to investigate the causal effect of 1,167 plasma proteins on osteonecrosis risk and identify potential therapeutic targets. Additionally, we conducted a phenome-wide MR (PheWAS-MR) analysis examine the side effects of the relevant proteins.

## Materials and methods

### Study design

**FIGURE 1:**
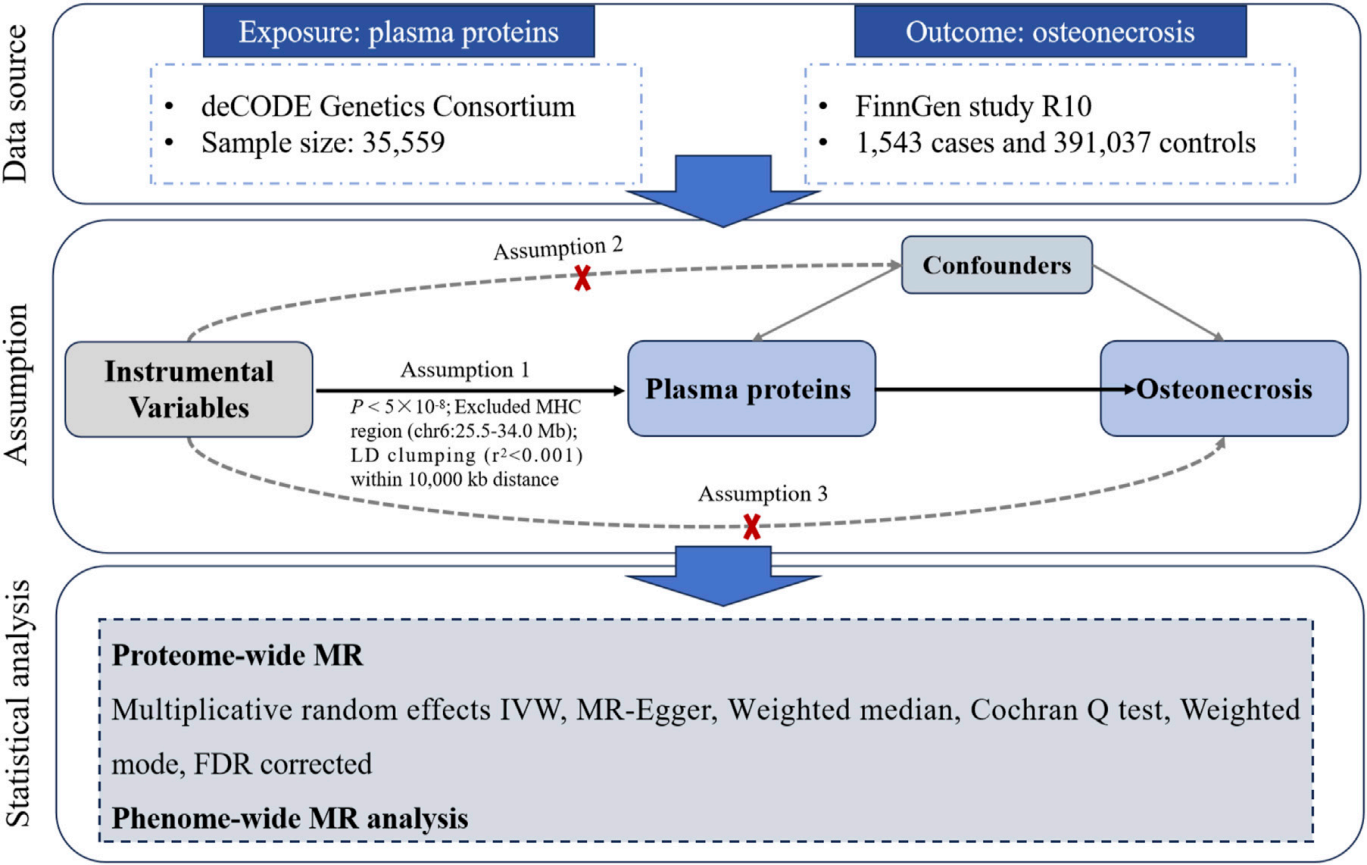
Overview of the study design in this study.

We performed a proteome-wide MR study to explore the causal association between 4,907 unique proteins (cis-pQTL) and osteonecrosis risk. This investigation was guided by three key principles: (1) the relevance criterion, ensuring that the instrumental variables (IVs) exhibit significant associations with the exposure variables (Davies et al., 2018); (2) the independence criterion, affirming that the IVs remain unaffected by any potential confounders, whether known or unknown (Davies et al., 2018) (3) the exclusion restriction criterion asserts that IVs affect the outcome exclusively through the exposure entities (Davies et al., 2018). Figure 1 illustrates the comprehensive framework of our analytical methodology. In short, we leveraged pQTL data from an extensive proteomic investigation and investigated their associations with osteonecrosis using MR analysis. Additionally, PheWAS-MR analyses were conducted to assess the druggability of identified protein biomarkers and prioritize therapeutic targets. This study utilized anonymized and publicly available datasets, informed consent or ethical review from an institutional board was not required.

### Data source

The plasma proteome data originated from a comprehensive GWAS by the deCODE Genetics Consortium (Ferkingstad et al., 2021). This study involved measuring 4,907 plasma protein levels in 35,559 Icelandic individuals using the SomaScan multiplex aptamer assay (Ferkingstad et al., 2021). Adjustments were made for age and sex and data were standardized by rank-inverse normal transformation (Ferkingstad et al., 2021). Specifically, the analysis employed the SomaScan version four assay (SomaLogic) to explore associations between 5,284 aptamers measuring 4,907 proteins and 27.2 million genetic variations, identifying significant links between 28,191 pQTLs and 4,631 proteins (Supplementary Table S1) (Ferkingstad et al., 2021). The protein-associated SNP associations with osteonecrosis were derived from the FinnGen study, a large-scale genomics initiative analyzing genetics and health data from over 500,000 Finnish biobank samples to elucidate disease mechanisms and genetic predispositions (Kurki et al., 2023). Utilizing the latest released data from the FinnGen study R10, which encompassed osteonecrosis-related diseases and included 1,543 cases and 391,037 controls (Supplementary Table S1) (Kurki et al., 2023).

### Selection of instrumental variables

For our analysis, we acquired data on 4,907 plasma proteins from the deCODE Genetics Consortium and applied three MR hypotheses to select instrumental variables (IVs). Initially, SNPs were identified with a significance level of *p* < 5 × 10^−8^. Notably, to account for the complex linkage disequilibrium (LD) patterns in the human major histocompatibility complex (MHC) region, SNPs in this area on chromosome 6 (spanning from 28477,897 to 33448,354) were excluded (Sun et al., 2023; Yuan et al., 2023). Cis-SNPs, considered to have a more direct and specific impact on protein function, were used as IVs in MR analyses, defined as those within 1 Mb of the gene encoding the respective protein (Sun et al., 2023; Yuan et al., 2023). The LD threshold parameter (r^2^) was set to 0.001 and the genetic distance was set to 10,000 kb to mitigate the impact of LD on independent SNPs. Subsequently, following the data harmonization between pQTLs and osteonecrosis, we omitted SNPs with *p* < 5 × 10^−8^ for osteonecrosis (Sun et al., 2023; Yuan et al., 2023). This exclusion was necessary to avoid strong correlations between these SNPs and osteonecrosis that could potentially compromise the accuracy of our results.

### MR analysis

The major method for determining causation was inverse-variance weighting (IVW) regression using a multiplicative random effects framework (Sun et al., 2023). The IVW approach gives high-power findings assuming that all IVs are authentic (Burgess et al., 2013). After extracting the association estimates linking the instruments and outcomes, and aligning the directional orientation of these estimates with the effect alleles, we utilized the Wald estimator to compute MR estimates for each instrument (Burgess et al., 2013). This approach enabled us to derive estimates of the causal effect. The findings from IVW method are considered reliable if each SNP adheres to the MR assumptions and is free from horizontal pleiotropy (Burgess et al., 2013). Cochran’s Q test was employed to evaluate heterogeneity among estimates from individual SNP. In the absence of heterogeneity, a fixed-effects model was applied, whereas the presence of heterogeneity necessitated the use of a random-effects model to provide more robust and reliable estimations (Greco et al., 2015). If heterogeneity was no significant (*p* < 0.05), a fixed-effects model was used; otherwise, a random-effects model was employed to provide a more reliable estimate (Greco et al., 2015). To enhance the validity of our results, we employed three MR methods. We also performed a sensitivity analysis to investigate horizontal pleiotropy using the MR-Egger regression intercept (Bowden et al., 2015). Regardless of the reliability of IVs, the MR-Egger technique gives a dependable approximation by computing causal effects using Egger regression’s slope coefficient (Bowden et al., 2015). Additionally, the weighted median method is recognized for its ability to control bias and minimize the risk of type I errors, providing consistent results even under less stringent IV assumptions (Bowden et al., 2016). For addressing horizontal pleiotropy, the MR-PRESSO global test and MR-Egger regression were primarily used, with a significance (Verbanck et al., 2018).

All statistical analyses were conducted using the “TwoSampleMR” packages in R version 4.1.2 (Hemani et al., 2018). Considering repetitive calculations, we took steps to reduce the possibility of incorrect findings in all MR investigations by employing the false discovery rate (FDR) method for *p*-value correction. A *P*_FDR_ value below 0.05 was considered statistically significant.

### Phenome-wide MR analysis

Phenome-wide association studies play a crucial role in drug development, aiding in the elucidation of mechanisms of action, identification of alternative indications, and prediction of adverse drug events (ADEs) (Yagensky et al., 2019). To further assess potential drug targets and potential ADEs, PheWAS-MR analysis was conducted on significant proteins associated with osteonecrosis, incorporating a wide array of disease phenotypes. To avoid redundancy in the data, we utilized the FinnGen R10 cohort (https://r10.finngen.fi/), comprising 412,181 participants. Disease outcomes were categorized using “PheCodes” to facilitate systematic genetic analysis of disease traits (Lee et al., 2019). Conditions with fewer than 50 cases were excluded, yielding 2,406 diseases for PheWAS-MR analyses. The PheWAS-MR results provide on the protective or risk factors associated with each standard deviation increase in plasma protein levels.

## Results

### Proteome-wide Mendelian randomization analysis

**FIGURE 2:**
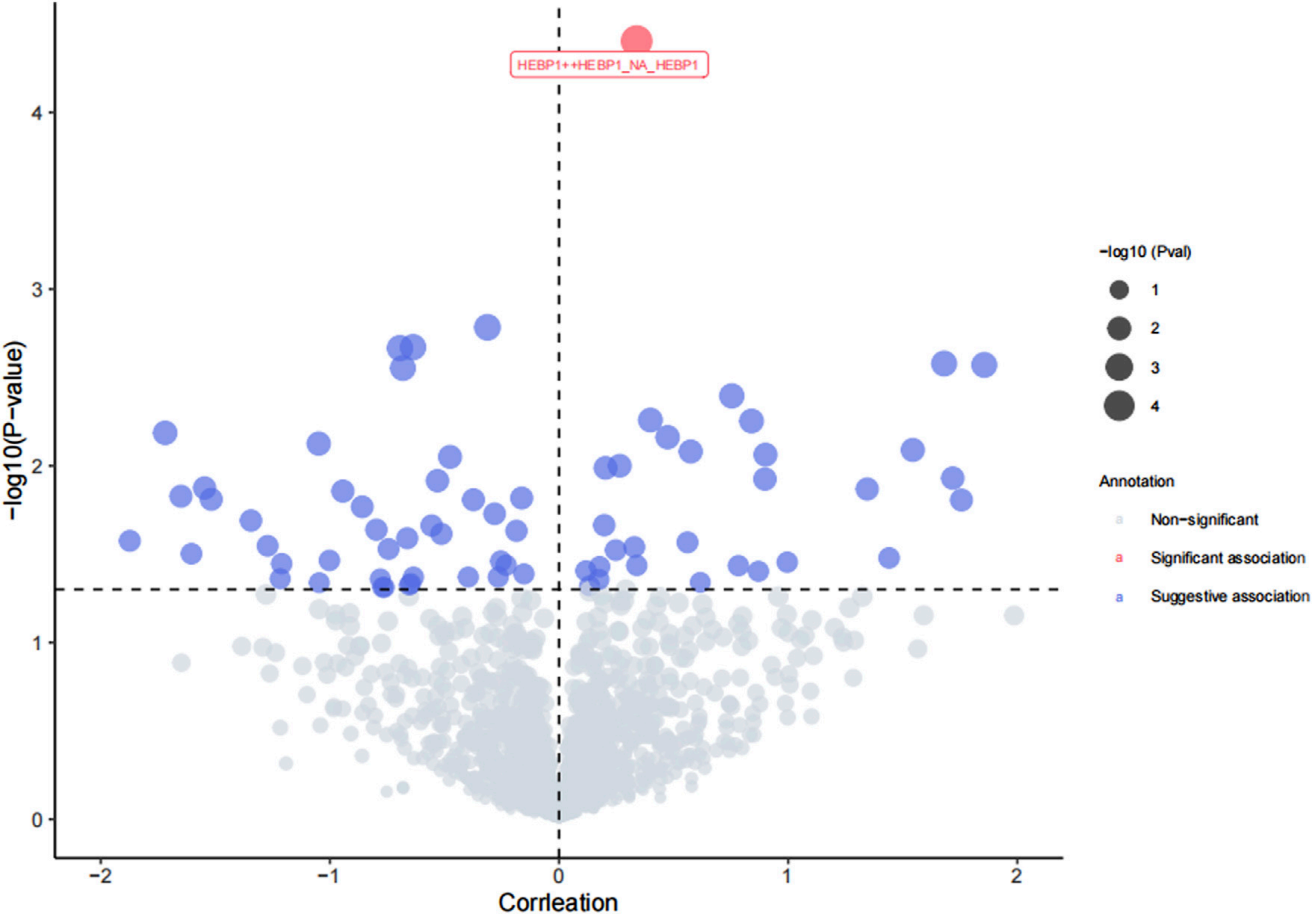
Volcano plot of MR results: Causal relationship between HEBP1 and osteonecrosis.

**FIGURE 3:**
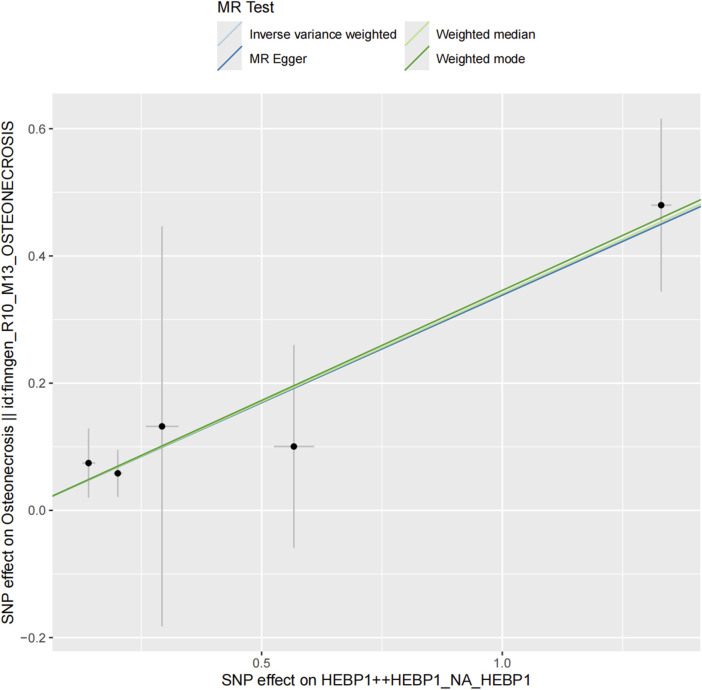
Association of genetically predicted Plasma Proteins with risk of Osteonecrosis.

We assessed the association between the risk of osteonecrosis and 1,676 plasma proteins with 5,517 genetic variants from the deCODE dataset. The F-value for these variants range from 31.49 to 15901.72, which is greater than 10, indicating that the instrument is not weak (Supplementary Table S2). We identified a total of 71 plasma proteins that are causally associated with osteonecrosis risk (Figure 2, *p* < 0.05). To avoid the potential type I error, we further conducted the FDR method to adjust the *p*-value and found that Heme-binding protein 1 (HEBP1) was positively associated with osteonecrosis risk (OR = 1.40, 95% CI, 1.19 to 1.65, *p* = 3.96 × 10^−5^, *P*_FDR_ = 0.044) (Figure 3). There was no heterogeneity among these IVs linked to HEBP1 (*p* = 0.950). Meanwhile, more evidence demonstrating the effect of HEBP1 on osteonecrosis risk was obtained using MR-Egger, weighted median, and weighted mode (Table 1). No pleiotropy of this association was observed in the MR-Egger (*p* = 0.993). Besides, we also found the positive association between Aldehyde dehydrogenase 3A1 (ALDH3A1) and osteonecrosis risk (Supplementary Table S3).

**TABLE 1:** The causal association between HEBP1 and osteonecrosis risk.

Method	Number of SNP	OR	LCI	UCI	*p*-Value	*P* For heterogeneity	*P* For pleiotropy
IVW	5	1.40	1.19	1.65	3.96E-05	0.871	
MR Egger	5	1.40	1.12	1.76	6.25E-02		0.993
Weighted median	5	1.41	1.18	1.67	1.23E-04		
Weighted mode	5	1.41	1.17	1.71	2.28E-02		

Additionally, we also found a suggestive association of inter-alpha trypsin inhibitor heavy chain 1 (ITIH1), secreted modular calcium-binding protein 1 (SMOC1), and cellular repressor of E1A-stimulated genes 1 (CREG1) proteins with osteonecrosis risk. These three plasma proteins had a negative effect on osteonecrosis risk with 0.73 (95% CI, 0.60–0.89), 0.50 (95% CI, 0.32–0.78), and 0.51 (95% CI, 0.32–0.79), respectively. More details are shown in Supplementary Tables S3-5.

### Phenome-wide MR analysis

**FIGURE 4:**
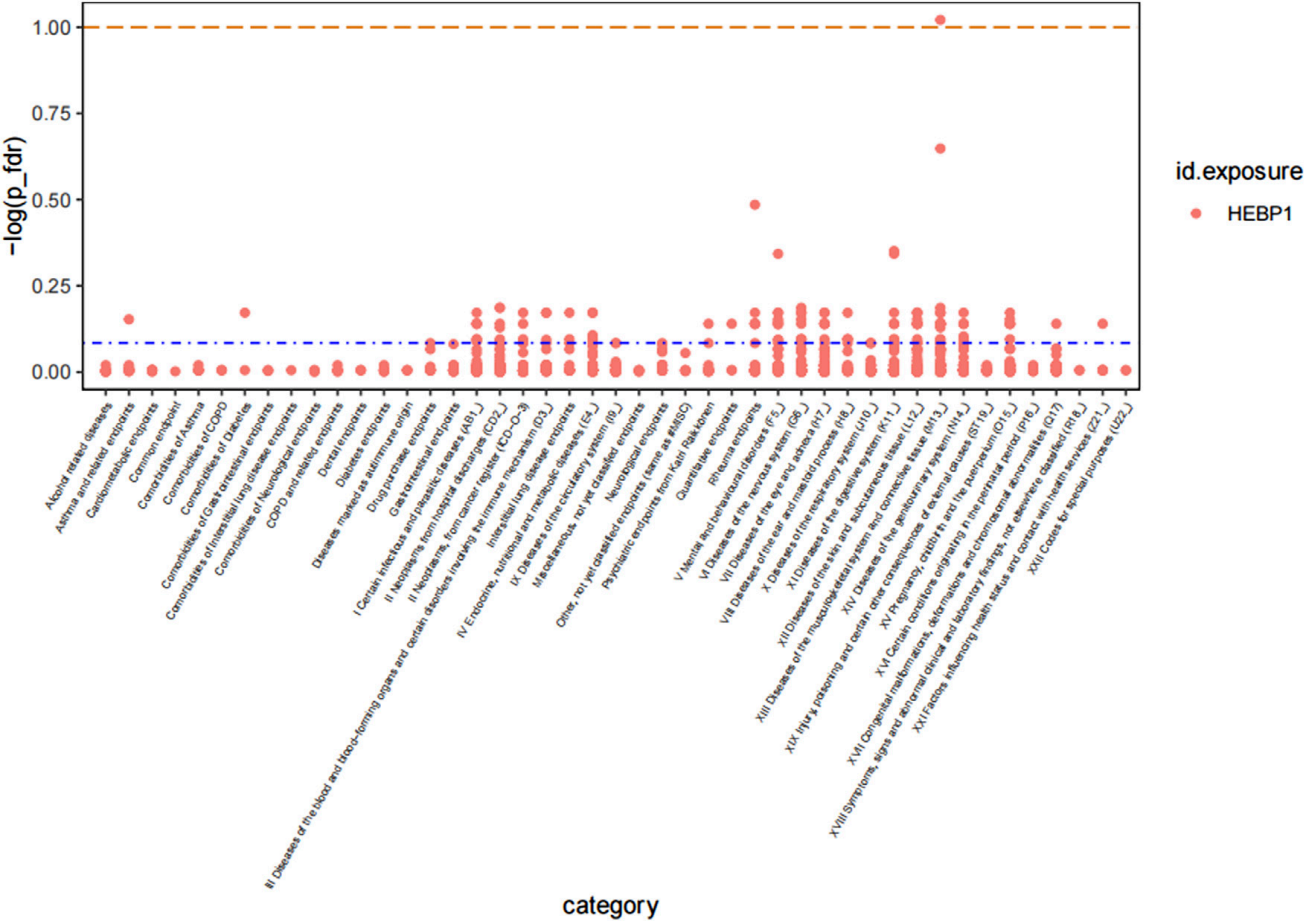
Manhattan plot of result of PheWAS analysis of associations between HEBP1 and osteonecrosis.

To comprehensively explore the health effect of HEBP1, which was found to be associated with osteonecrosis risk, we conducted a PheWAS-MR association analysis, screening 2,406 traits from the Finnish GWAS (version 10). Our findings revealed that HEBP1 was associated with 136 phenotypes excluding osteonecrosis (*p* < 0.05; Figure 4). For instance, HEBP1 was associated with decreased risk of other secondary gout (OR, 0.34, 95% CI, 0.18 to 0.61, *p* = 4.08 × 10^−4^) and hallux rigidus (OR, 0.88, 95% CI, 0.81 to 0.95, *p* = 2.16 × 10^−3^). Particularly noteworthy was the risk effect of HEBP1 on clinical traits such as tooth eruption problems, emotionally unstable personality disorder, and encephalopathy (Supplementary Table S6). However, these associations did not reach a significant level after the FDR adjustment, indicating that the plausibility is suggestive.

## Discussion

This study based on large-scale GWAS data is the first to utilize comprehensive MR analysis, and PheWAS-MR analysis to explore the potential causal relationships between plasma proteins and osteonecrosis risk. Our findings reveal that genetically predicted levels of 30 plasma proteins exhibit a significant positive association with osteonecrosis, while 41 plasma proteins show a significant negative association. These findings suggest that the identified plasma proteins could be valuable for the early diagnosis, treatment, and prevention of osteonecrosis.

In this study, we identified 30 plasma proteins positively associated with osteonecrosis, and in particular, HEBP1 significantly increased the risk of osteonecrosis and ALDH3A1 suggestive increased the risk of osteonecrosis. HEBP1 is an intracellular tetrapyrrole-binding protein potentially involved in biosynthesis of heme or porphyrin (Taketani et al., 1998). Heme plays a key role in oxygen-binding and transport molecules such as hemoglobin and myoglobin (Immenschuh et al., 2017). Recent studies have shown the importance of HEBP1 in the central nervous system, particularly in neurodegenerative diseases (Yagensky et al., 2019; Chua, 2023). However, our study identifies a positive association between HEBP1 and osteonecrosis risk. This is consistent with a case-control study that found the HEBP1 *gene* in bisphosphonate-induced osteonecrosis of jaw patients (Lee et al., 2019). This gene encodes HBP1, and heme is a complex of iron and tetrapyrrole protoporphyrin IX, which is the prosthetic group in hemoproteins. Hemoproteins play a key role in oxygen binding and the transportation of compounds such as hemoglobin and myoglobin. Another study also found that HEBP1 expression levels significantly affected the development of bone metastasis from breast cancer (Park et al., 2018). ALDH3A1, part of aldehyde dehydrogenase family, plays a role in various cellular processes such as lipid metabolism, drug metabolism, and oxidative stress response (Pappa et al., 2003; Muzio et al., 2012; Voulgaridou et al., 2020). Oxidative stress may deteriorate osteoarthritis of the temporomandibular joint function, leading to the degradation of collagen polysaccharides and the activation of enzymes that contribute to cartilage breakdown (Roberts et al., 1989; Kawai et al., 2008). Despite the lack of pharmacological information on HEBP1 and ALDH3A1, it remains a promising prognostic biomarker and therapeutic marker for osteonecrosis. Future studies further validate the association by examining whether anti-HEBP1/ALDH3A1 therapeutic antibodies have a role in osteonecrosis.

We also noticed 41 plasma proteins suggestive negatively associated with osteonecrosis. We have specifically focused on the roles of ITIH1, SMOC1, and CREG1 proteins. These proteins offer valuable insights into the protective mechanisms that could counteract the development of osteonecrosis. ITIH1, a glycoprotein from the inter-α-trypsin inhibitor (IαI) family, covalently attaches to hyaluronic acid molecules through its heavy chains (Chen et al., 1994). Although limited research exists on the association between ITIH1 and osteonecrosis, previous studies have linked ITIH1 with osteoarthritis (Chen et al., 2015; Lourido et al., 2017; Lourido et al., 2021). Proteomic analysis revealed significantly higher levels of ITIH1 in knee osteoarthritis patients than in healthy controls (Lourido et al., 2017). Another study indicates that ITIH1 may enhance the ability to predict the incidence of knee osteoarthritis in clinical practice (Lourido et al., 2021). In addition, Chen et al. found a significant reduction in ITIH4 in steroid-induced ONFH in adults (Chen et al., 2015).

The association between SMOC1 and osteonecrosis risk has not been investigated. However, there is some indirect evidence suggesting SMOC1 is associated with bone formation (Choi et al., 2010; Takahata et al., 2021). For example, experimental evidence from mice indicates that SMOC1 and SMOC2 are novel targets of Runx2, and they are pivotal in both intramembranous and endochondral bone formation processes (Takahata et al., 2021). Runx2 is a crucial transcription factor that regulates bone formation (Lin et al., 2011; Xu et al., 2020). Another experimental study showed SMOC1 is an important extracellular matrix protein in osteoblast differentiation (Choi et al., 2010). Moreover, an *in vitro* study validated the secretion of angiogenic proteins by human pluripotent mesenchymal stromal cells, which may improve the efficiency of surgical interventions for osteonecrosis (Müller et al., 2008). These experimental and population studies imply that SMOC1 may be a potential target for osteonecrosis therapy.

Previous studies have identified CREG1 as a protein involved in cellular differentiation and homeostasis, with expressed across various tissues, including the spleen, liver, kidney, lung, heart, fat tissue, and skeletal muscle. Our study found that CREG1 reduces the risk of developing osteonecrosis, which is consistent with other findings. A study of CREG1 knockdown by adeno-associated virus impeded myogenic differentiation and skeletal muscle regeneration after injury *in vivo*, whereas CREG1 overexpression in muscle satellite cells accelerated the process of CTX-induced skeletal muscle regeneration (Song et al., 2024). CREG1 improves the capacity of the skeletal muscle response to exercise endurance via modulation of mitophagy (Song et al., 2021). These studies show that CREG1 positively regulates skeletal muscle regeneration, indicating its potential as a therapeutic target for enhancing muscle regeneration.

This comprehensive MR study identified 71 genetically predicted plasma proteins significantly associated with osteonecrosis risk. Among these, 41 proteins were downregulated, and 30 proteins were upregulated, indicating their involvement in the development of osteonecrosis. The pathogenesis of osteonecrosis has not been fully elucidated, however, the majority of these proteins are associated with current models of osteonecrosis etiology based on three main pillars (Malizos et al., 2007; Chang et al., 2020; Kalita et al., 2023; Meng et al., 2023): 1) inhibition of the remodeling and resorption of osteoclastic bone. 2) angiogenesis supperssion, and 3) inflammation and infection. Although many plasma proteins were not discussed in this study, it does not imply that they are unimportant in the development of osteonecrosis. Further studies are required to validate their role in the future.

The strengths of this study are that it explores the causal associations between several plasma proteins and osteonecrosis at the gene level, identifies plasma proteins significantly associated with osteonecrosis, and highlighting the public health importance of this finding for future diagnosis and treatment of osteonecrosis. Secondly, this study was based on large-scale GWAS data with a large sample sizes and high statistical power. Multiple MR analyses were also used, combining Q-test and Egger regression to assess the likelihood of heterogeneity and directional selection. The results of these analyses indicated that our findings were robust and not affected by horizontal pleiotropy or confounding factors. However, our study has limitations. Firstly, this study is based on a European database and may not apply to other ethnic groups. Secondly, this study lacks plasma protein level data in other tissues, which hinders the association between plasma protein levels in other tissues and osteonecrosis risk. Additionally, a more comprehensive GWAS database and advanced analytical methods or experimental validation are needed to clarify the associations between individual plasma proteins and osteonecrosis and their mechanisms. Lastly, the practical application value of candidate plasma proteins needs validation through comprehensive clinical trials. Future studies could investigate targeted therapy mechanisms for osteonecrosis.

## Conclusion

In conclusion, this study demonstrated a causal link between multiple plasma proteins and osteonecrosis through a comprehensive MR analysis, especially HEBP1*,* which provides a new pathway for the biological mechanism of osteonecrosis and helps to explore early intervention and treatment. Nonetheless, further studies are needed to validate this candidate plasma protein.

## Data Availability

The original contributions presented in the study are included in the article/Supplementary Material, further inquiries can be directed to the corresponding authors.
